# The triglyceride-glucose index is a predictor of major adverse cardiovascular events in patients with coronary artery disease and psoriasis: a retrospective cohort study

**DOI:** 10.1186/s13098-024-01423-8

**Published:** 2024-07-31

**Authors:** Bingqi Fu, Yan Zeng, Man Wang, Lin Zhao, Lin Sun, Tianjie Wang, Junle Dong, Weixian Yang, Wei Hua

**Affiliations:** 1 Department of Cardiology, Fuwai Hospital, National Center for Cardiovascular Disease, Chinese Academy of Medical Sciences and Peking Union Medical College, No. 167, Beilishi Road, Beijing, 100037 China; 2 Department of Integrative Medicine Cardiology, China-Japan Friendship Hospital, Beijing, 100029 China

**Keywords:** Triglyceride-glucose index, Coronary artery disease, Psoriasis, MACE

## Abstract

**Background:**

The association between the triglyceride-glucose (TyG) index and clinical outcomes in patients with both coronary artery disease (CAD) and psoriasis is unclear. This study investigated the association between the TyG index and major adverse cardiovascular events (MACE) in patients with both CAD and psoriasis.

**Methods:**

This retrospective cohort study included patients diagnosed with both CAD and psoriasis who underwent coronary angiography at the Fuwai Hospital, Beijing, China, between January 2017 and May 2022. The study endpoint was the occurrence of MACE or end of follow-up time. Multivariate Cox proportional analysis and restricted cubic splines (RCS) were used to determine the association between the TyG index and MACE. Receiver operating characteristic (ROC) curves were used to determine the optimal threshold value of the TyG index for predicting MACE.

**Results:**

This study enrolled 293 patients with both CAD and psoriasis, including 258 (88.1%) males with a mean age of 58.89 ± 9.61 years. Patients were divided into four groups based on the TyG quartiles: Q1 (*N* = 74), Q2 (*N* = 73), Q3 (*N* = 73), and Q4 (*N* = 73). After adjusting for the potential confounders, the TyG index was independently associated with MACE, both as a continuous variable (HR = 1.53, 95% CI = 1.03–2.28, *P* = 0.035) and as a categorical variable (Q1: reference; Q2: HR = 1.85, 95% CI = 0.88–3.87, *P* = 0.105; Q3: HR = 2.39, 95% CI = 1.14-5.00, *P* = 0.021; Q4: HR = 2.19, 95% CI = 1.001–4.81, *P* = 0.0497; P for trend = 0.039). RCS analysis showed an linear association between the TyG index and MACE (P-overall = 0.027, P-non-linear = 0.589). ROC curve analysis showed that the TyG index of ≥ 8.73 was the optimal threshold value (area under the ROC curve = 0.60, 95% CI 0.53–0.67). TyG index ≥ 8.73 was significantly associated with MACE (HR = 2.10, 95% CI = 1.32–3.34, *P* = 0.002). After adjustment for confounders, the TyG index showed independent association with MACE (HR = 2.00, 95% CI = 1.17–3.42, *P* = 0.011).

**Conclusions:**

The TyG index showed a positive linear correlation with MACE in patients with both CAD and psoriasis. The TyG index of ≥ 8.73 might be the optimal threshold for predicting MACE.

**Graphical Abstract:**

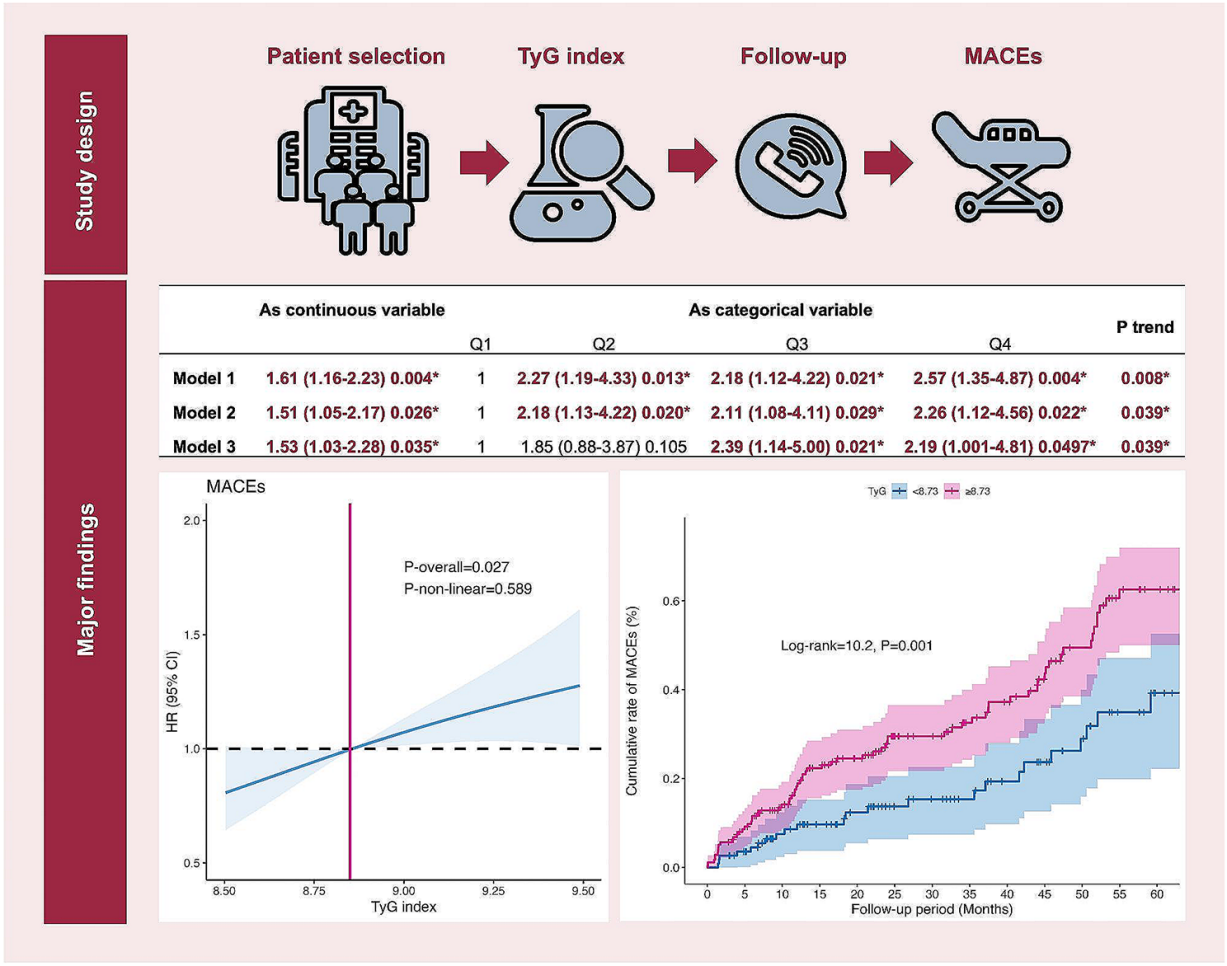

## Introduction

Coronary artery disease (CAD) is the most common form of cardiovascular disease and affects nearly half of the adult population in the United States [1, 2]. Psoriasis is a chronic systemic inflammatory disease that affects approximately 3% of the US population [3] and increases the risk of cardiovascular diseases [4, 5]. Previous studies have reported that psoriasis was significantly associated with an higher incidence of CAD [6, 7] and myocardial infarction [8, 9]. This may be caused by the common genetic mechanisms that play a pathogenic role in both CAD and psoriasis [10, 11]. Both diseases involve chronic inflammation, which damages both the vascular endothelium and the skin epithelium. The inherent risk of cardiovascular complications in the psoriasis patients necessitates proactive screening for the early identification of high-risk patients [4].

The triglyceride-glucose (TyG) index is a measure of insulin resistance (IR) and has been extensively validated as an effective predictor of adverse cardiovascular events subjects with CAD [12]. TyG index is also an indicator of systemic inflammation [13]. Cross-sectional studies have suggested a potential correlation between the TyG index and the occurrence of psoriasis [14], and the incidence of carotid atherosclerosis in psoriatic arthritis [15]. Although several studies have reported that the TyG index is a potential biomarker for predicting cardiovascular outcomes, its predictive value in patients with both CAD and psoriasis remains unclear. Therefore, this study investigated the association between the TyG index and major adverse cardiovascular events (MACE) in patients with both CAD and psoriasis.

## Materials and methods

### Ethic approval and study population

This retrospective cohort study was conducted in accordance with the Declaration of Helsinki guidelines and approved by the Ethics Committee of Fuwai Hospital (Approval No. 2021–1544). Written informed consent was obtained from all the study participants.

**Fig. 1:**
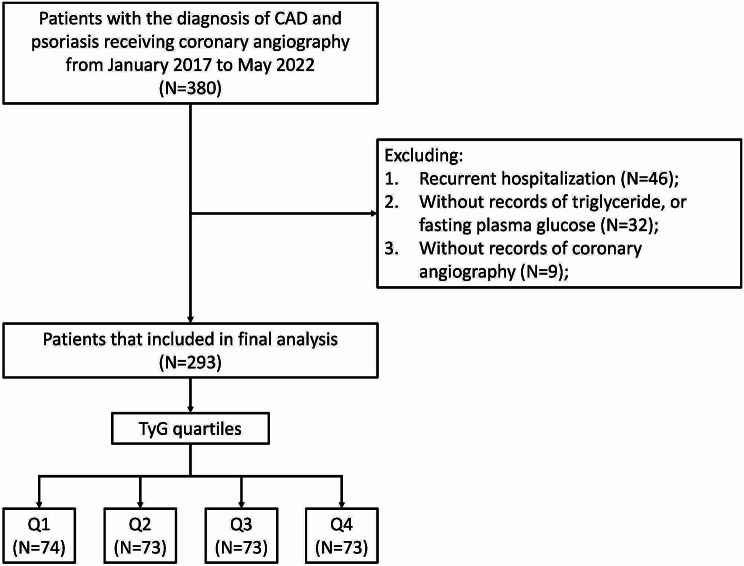
Flowchart of study population. *Abbreviations* TyG index, triglyceride-glucose index; CAD, coronary artery disease

We consecutively enrolled patients diagnosed with CAD and psoriasis that received coronary angiography at the Fuwai Hospital, Beijing, China, between January 2017 and May 2022. We excluded patients with recurrent hospitalization (*N* = 46), patients without records of fasting triglyceride or fasting plasma glucose (*N* = 32), and patients without records of coronary angiography (*N* = 9). Subsequently, 293 patients were included in the final analysis (Fig. 1).

### Measurement of the TyG index

Fasting blood samples were collected by trained nurses from all enrolled patients during admission. Fasting triglyceride and fasting plasma glucose concentrations were measured using standard biochemical techniques in the core laboratory at the Fuwai Hospital. The TyG index was calculated using the following formula: Ln [fasting triglyceride (mg/dL) × fasting plasma glucose (mg/dL)/2]. Patients were categorized into the following four groups based on the TyG index quartiles: Q1 (*N* = 74), Q2 (*N* = 73), Q3 (*N* = 73), and Q4 (*N* = 73). Patients in the Q1 group were set as the reference group (Fig. 1).

### Data collection and definitions

Trained study coordinators extracted data from the electronic medical recording system, including demographic information, current and past medical history, medical therapy, laboratory results, echocardiography, coronary angiography, and treatment strategies.

The diagnosis of psoriasis was confirmed by a dermatologist through clinical examination of skin lesions or histological analysis from a biopsy [5]. Patients reported their ongoing psoriasis treatments. Psoriasis Area and Severity Index (PASI) was used to estimate the severity and extent of psoriasis. Psoriasis treatments included non-biologic therapies such as steroids and methotrexate [16] and biologic treatments such as inhibitors of interleukin 12/23, tumor necrosis factor-alpha, and interleukin 17 [17].

Patients admitted with acute coronary syndrome (ACS) presented with a primary diagnosis of ST-segment elevation myocardial infarction, non-ST-segment elevation myocardial infarction, or unstable angina [18, 19]. Coronary angiography was performed by experienced surgeons and the lesion morphology was assessed by two independent surgeons. Left main or three-vessel disease (LM/TVD) was defined by the angiographic evidence of ≥ 50% stenosis in the three primary epicardial coronary arteries—left anterior descending, left circumflex, and right coronary arteries, with or without involvement of the left main artery [20]. Treatment strategies included medical therapy, percutaneous coronary intervention (PCI), or coronary artery bypass grafting (CABG), and were performed according to the current practice guidelines, judgement of the cardiologists, and patient preferences.

### Follow-up and study endpoints

All the patients were followed up through telephone interviews, or outpatient clinical visits. The endpoint of the study was MACE, which was defined as a composite of cardiac death, ACS, stroke, urgent revascularization, and heart failure. The duration of follow-up was estimated from the date of hospitalization to the date of the occurrence of MACE or the date of the follow-up deadline (March 1st, 2024). The median follow-up time was 32.8 months.

### Statistical analysis

The continuous variables were presented as the mean ± standard deviation or median (25th quartile, 75th quartile) and analyzed using the Analysis of Variance or the Kruskal-Wallis Rank Test. The categorical variables were presented as frequency (percentage) and analyzed using the chi-squared test.

Univariate Cox proportional hazard regression analysis was performed to identify the potential risk factors for MACE. Then, significant risk factors from the univariate Cox regression model (Model 1) were included in the multivariate Cox regression models (Model 2 and Model 3) along with covariables such as age, sex, smoking status, hypertension, type 2 diabetes mellitus (T2DM), stroke, admission for ACS, use of renin-angiotensin-aldosterone system inhibitors (RAASI), platelet count, high sensitivity C-reactive protein (hsCRP), low-density lipoprotein cholesterol (LDL-C), left ventricular ejection fraction (LVEF), LM/TVD, angulated lesion, and PCI therapy, to determine whether the TyG index was an independent risk factor for predicting MACE. The trend of increasing hazard ratios (HRs) with the TyG index was evaluated by calculating the P value for trend.

The relationship between the TyG index and MACE was further analyzed using the Cox proportional hazard regression models with restricted cubic splines (RCS) and smooth curve fitting based on the penalized spline method. If a linear relationship was observed, the threshold value was estimated using the Receiver Operating Characteristics (ROC) curves, and the optimal threshold was considered as the point at which the Youden index was maximized. Kaplan-Meier curves were plotted to compare the cumulative rates of MACE during the follow-up between the higher and lower TyG index groups, which were categorized based on the threshold TyG index. The differences in the survival rates between the groups were compared using the log-rank test. We also performed stratified analyses based on age (< 60 years or ≥ 60 years), gender, smoking status, hypertension, T2DM, admission for ACS, PCI therapy, LM/TVD, and LVEF (< 50%, or ≥ 50%). P interaction values were calculated to determine the interaction between the TyG index and the stratified variables. R software version 4.3.1 was used to perform the statistical analyses. Two-tailed *P* < 0.05 was considered statistically significant.

## Results

### Baseline clinical characteristics

This study included 293 patients with both CAD and psoriasis. The baseline characteristics of the study subjects are summarized in Table 1. The mean age of the study subjects was 58.89 ± 9.61 years and 258 out of 293 (88.1%) patients were male. The average duration of psoriasis was 23.67 ± 11.94 years, and 126 (43.0%) patients received topical therapy. The median PASI was 6.3 (0.8, 13.4). Evaluation of the coronary arterial characteristics demonstrated LM/TVD in 44 (15.0%) patients. Evaluation of the coronary lesion morphology showed angulated lesions in 52 patients (17.7%), bifurcation lesions in 49 patients (16.7%), chronic total occlusions in 31 patients (10.6%), and ostial lesions in 26 patients (8.9%). Thrombus formation was presented by only 5 (1.7%) patients. Patients were divided into four quartiles based on the TyG index. Patients in the highest quartile were associated with significantly higher prevalence of T2DM, metformin use, treatment with PCI, and the occurrence of MACE (all *P* < 0.05).

**Table 1:** Baseline characteristics

Characteristics	Total (*N* = 293)	Q1 (*N* = 74)	Q2 (*N* = 73)	Q3 (*N* = 73)	Q4 (*N* = 73)	*P* value
		7.43–8.48	8.48–8.86	8.86–9.32	9.32–10.90	
Age	58.89 ± 9.61	60.49 ± 10.02	58.25 ± 10.32	59.82 ± 8.54	56.99 ± 9.26	0.115
Male gender	258 (88.1)	67 (90.5)	66 (90.4)	62 (84.9)	63 (86.3)	0.635
Smoke	197 (67.2)	45 (60.8)	55 (75.3)	46 (63.0)	51 (69.9)	0.223
**Admission for ACS**	171 (58.4)	36 (48.6)	41 (56.2)	47 (64.4)	47 (64.4)	0.158
**Past medical history**						
HTN	174 (59.4)	38 (51.4)	46 (63.0)	40 (54.8)	50 (68.5)	0.137
T2DM	105 (35.8)	16 (21.6)	23 (31.5)	18 (24.7)	48 (65.8)	< 0.001
Stroke	27 (9.2)	3 (4.1)	7 (9.6)	7 (9.6)	10 (13.7)	0.247
**Psoriasis characteristics**						
Disease duration	23.67 ± 11.94	24.31 ± 11.87	24.40 ± 11.39	23.97 ± 13.69	22.06 ± 10.96	0.641
Psoriatic arthritis	9 (3.1)	3 (4.1)	1 (1.4)	3 (4.1)	2 (2.7)	0.743
PASI	6.3 (0.8, 13.4)	5.5 (0, 12.5)	6.3 (2, 14.4)	7.2 (1.0, 11.7)	6 (0.8, 14.3)	0.778
Topical therapy	126 (43.0)	26 (35.1)	33 (45.2)	32 (43.8)	35 (47.9)	0.430
Phototherapy	21 (7.2)	4 (5.4)	4 (5.5)	3 (4.1)	10 (13.7)	0.095
Biologic therapy	26 (8.9)	6 (8.1)	8 (11.0)	5 (6.8)	7 (9.6)	0.835
Non-biologic therapy	83 (28.3)	26 (35.1)	18 (24.7)	25 (34.2)	14 (19.2)	0.092
**Medical therapy**						
Aspirin	268 (91.5)	70 (94.6)	68 (93.2)	63 (86.3)	67 (91.8)	0.296
P2Y12 inhibitors	224 (76.5)	57 (77.0)	56 (76.7)	55 (75.3)	56 (76.7)	0.995
RAAS inhibitors	152 (51.9)	38 (51.4)	33 (45.2)	39 (53.4)	42 (57.5)	0.510
β blocker	246 (84.0)	60 (81.1)	63 (86.3)	59 (80.8)	64 (87.7)	0.566
Statin	280 (95.6)	71 (95.9)	69 (94.5)	68 (93.2)	72 (98.6)	0.418
Metformin	38 (13.0)	6 (8.1)	6 (8.2)	7 (9.6)	19 (26.0)	0.002
**Laboratory results**						
Platelet count	227.28 ± 63.48	223.55 ± 59.72	223.21 ± 62.31	229.38 ± 70.77	233.04 ± 61.37	0.746
Hemoglobin	146.13 ± 16.20	144.39 ± 16.91	145.23 ± 16.81	145.12 ± 14.36	149.81 ± 16.35	0.163
hsCRP	2.94 (1.77, 5.26)	2.45 (1.50, 3.77)	2.94 (1.66, 5.77)	3.19 (1.93, 6.02)	3.36 (2.05, 5.07)	0.234
Fasting glucose	6.94 ± 2.96	5.52 ± 1.14	6.04 ± 1.32	6.51 ± 1.95	9.72 ± 4.21	< 0.001
Triglyceride	1.63 ± 0.95	0.89 ± 0.22	1.30 ± 0.26	1.77 ± 0.43	2.59 ± 1.31	< 0.001
Total cholesterol	3.92 ± 0.97	3.63 ± 0.89	3.87 ± 0.90	4.09 ± 1.07	4.08 ± 0.95	0.012
LDL-C	2.33 ± 0.85	2.12 ± 0.78	2.33 ± 0.78	2.53 ± 0.96	2.34 ± 0.83	0.033
HDL-C	1.12 ± 0.34	1.19 ± 0.30	1.12 ± 0.26	1.13 ± 0.45	1.04 ± 0.29	0.065
**Echocardiography**						
LAD	37.44 ± 5.83	36.86 ± 5.71	38.44 ± 7.43	37.47 ± 5.19	36.97 ± 4.50	0.350
LVDD	49.44 ± 6.45	49.29 ± 7.25	50.99 ± 6.79	49.20 ± 6.43	48.20 ± 4.86	0.078
LVEF	59.94 ± 8.93	60.10 ± 10.81	58.00 ± 9.82	60.10 ± 7.55	61.62 ± 6.58	0.116
**Coronary angiography**						
LM/TVD	44 (15.0)	9 (12.2)	14 (19.2)	11 (15.1)	10 (13.7)	0.668
Chronic total occlusion	31 (10.6)	5 (6.8)	13 (17.8)	4 (5.6)	9 (12.3)	0.063
Angulated lesion	52 (17.7)	11 (14.9)	15 (20.5)	9 (12.3)	17 (23.3)	0.282
Ostial lesion	26 (8.9)	10 (13.5)	8 (11.0)	3 (4.1)	5 (6.8)	0.189
Bifurcation lesion	49 (16.7)	11 (14.9)	12 (16.4)	12 (16.4)	14 (19.2)	0.917
Thrombus presence	5 (1.7)	0 (0.0)	3 (4.1)	0 (0.0)	2 (2.7)	0.137
**Treatment**						
PCI	151 (51.5)	29 (39.2)	39 (53.4)	35 (47.9)	48 (65.8)	0.012
CABG	11 (3.8)	3 (4.1)	4 (5.5)	0 (0.0)	4 (5.5)	0.254
**MACEs**	94 (32.3)	14 (18.9)	27 (37.0)	24 (33.8)	29 (39.7)	0.033

*Abbreviations* ACS, acute coronary syndrome; HTN, hypertension; T2DM, type 2 diabetes mellitus; RAASI, renin-angiotensin-aldosterone system inhibitors; hsCRP, high sensitivity C-reactive protein; FPG, fasting plasma glucose; LDL-C, low-density lipoprotein cholesterol; HDL-C, high-density lipoprotein cholesterol; LAD, left atrium diameter; LVDD, left ventricle end-diastolic diameter; LVEF, left ventricular ejection fraction; LM/TVD, left main or three-vessel disease; PCI, percutaneous coronary intervention; CABG, coronary artery bypass grafting; MACEs, major adverse cardiovascular events

### Association between the TyG index and MACE

Ninety-four patients in the study cohort experienced MACE. Univariate Cox regression analysis demonstrated that the TyG index was significantly associated with MACE, both as a continuous variable (HR = 1.61, 95% CI = 1.16–2.23, *P* = 0.004; Table 2) and as a categorical variable (Q1: reference; Q2: HR = 2.27, 95% CI = 1.19–4.33, *P* = 0.013; Q3: HR = 2.18, 95% CI = 1.12–4.22, *P* = 0.021; Q4: HR = 2.57, 95% CI = 1.35–4.87, *P* = 0.004; P for trend = 0.008; Table 2).

**Table 2:** Cox regression analyses for the association between TyG index and MACEs

	As continuous variable	As categorical variable				
		Q1	Q2	Q3	Q4	*P* trend
Model 1	1.61 (1.16–2.23) 0.004	1	2.27 (1.19–4.33) 0.013	2.18 (1.12–4.22) 0.021	2.57 (1.35–4.87) 0.004	0.008
Model 2	1.51 (1.05–2.17) 0.026	1	2.18 (1.13–4.22) 0.020	2.11 (1.08–4.11) 0.029	2.26 (1.12–4.56) 0.022	0.039
Model 3	1.53 (1.03–2.28) 0.035	1	1.85 (0.88–3.87) 0.105	2.39 (1.14-5.00) 0.021	2.19 (1.001–4.81) 0.0497	0.039

Model 1: Non-adjusted

Model 2: adjusted for age, sex, smoking status, hypertension, T2DM, stroke, and admission for ACS

Model 3: adjusted for age, sex, smoking status, hypertension, T2DM, stroke, and admission for ACS, use of RAASI, platelet count, hsCRP levels, LDL-C, LVEF, presence of LM/TVD, angulated lesion, and PCI therapy

*Abbreviations* TyG, triglyceride-glucose; MACEs, major adverse cardiovascular events; T2DM, type 2 diabetes mellitus; ACS, acute coronary syndrome; RAASI, renin-angiotensin-aldosterone system inhibitors; hsCRP, high sensitivity C-reactive protein; LDL-C, low-density lipoprotein cholesterol; LVEF, left ventricular ejection fraction; LM/TVD, left main or three-vessel disease; PCI, percutaneous coronary intervention

Multivariate models were developed to assess the independent association of the TyG index with MACE. After adjusting for potential confounding factors, including age, sex, smoking status, hypertension, T2DM, stroke, and admission for ACS in Model 2, the TyG index showed independent association with MACE as a continuous variable (HR = 1.51, 95% CI = 1.05–2.17, *P* = 0.026; Table 2) and as a categorical variable (Q1: reference; Q2: HR = 2.18, 95% CI = 1.13–4.22, *P* = 0.020; Q3: HR = 2.11, 95% CI = 1.08–4.11, *P* = 0.029; Q4: HR = 2.26, 95% CI = 1.12–4.56, *P* = 0.022; P for trend = 0.039; Table 2).

In Model 3, we adjusted for confounding factors included in Model 2 as well as use of RAASI, platelet count, hsCRP levels, LDL-C, LVEF, presence of LM/TVD, angulated lesion, and PCI therapy. The results from Model 3 also showed that the TyG index was independently associated with MACE as a continuous variable (HR = 1.53, 95% CI = 1.03–2.28, *P* = 0.035; Table 2) and as a categorical variable (Q1: reference; Q2: HR = 1.85, 95% CI = 0.88–3.87, *P* = 0.105; Q3: HR = 2.39, 95% CI = 1.14-5.00, *P* = 0.021; Q4: HR = 2.19, 95% CI = 1.001–4.81, *P* = 0.0497; P for trend = 0.039; Table 2).

### Identification of the linear relationship between TyG index and MACE and determination of the optimal threshold for the TyG index

**Fig. 2:**
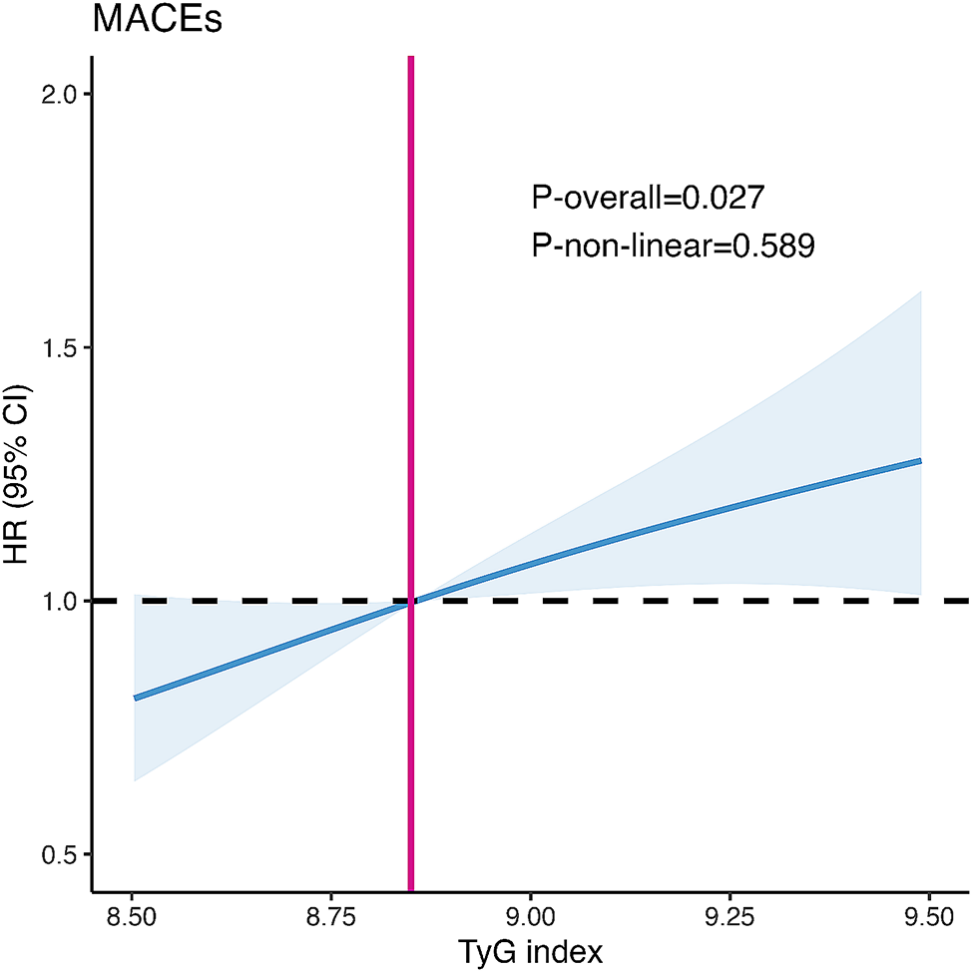
Restricted cubic splines for the association between TyG index and MACEs. *Abbreviations* TyG index, triglyceride-glucose index; MACEs, major adverse cardiovascular events; HR, hazard ratio

RCS analysis demonstrated a linear association between the TyG index and MACE (P-overall = 0.027, P-non-linear = 0.589; Fig. 2).

**Fig. 3:**
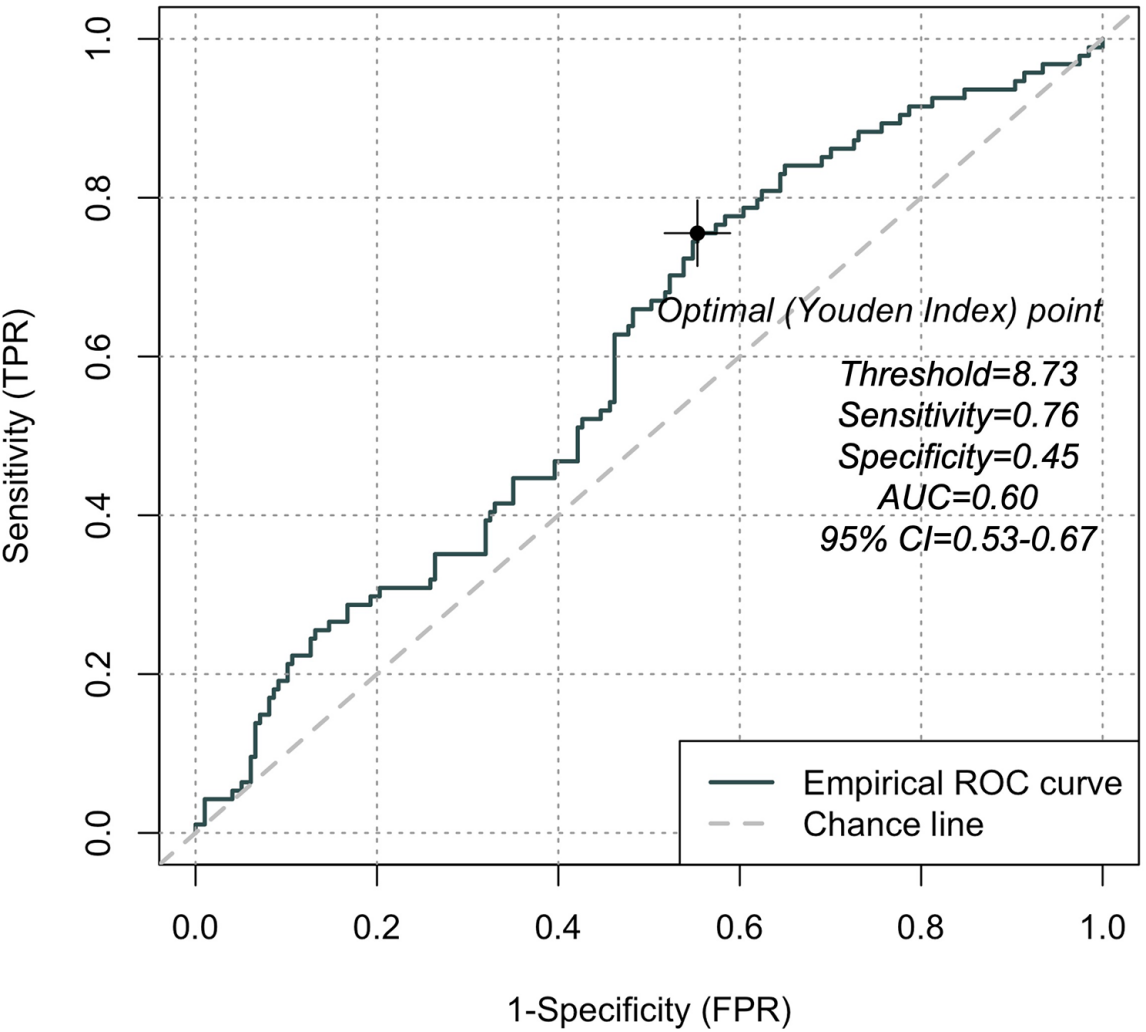
Receiver Operating Characteristic curve for TyG index in predicting MACEs

ROC curve analysis was performed to determine the optimal cutoff value of the TyG index for predicting MACE. ROC curve analysis results demonstrated that a TyG index of ≥ 8.73 was the optimal threshold with a sensitivity of 0.76, specificity of 0.45, and an area under the curve value of 0.60 (95% CI = 0.53–0.67) (Fig. 3).

### Evaluation of the optimal threshold and stratified analyses

**Fig. 4:**
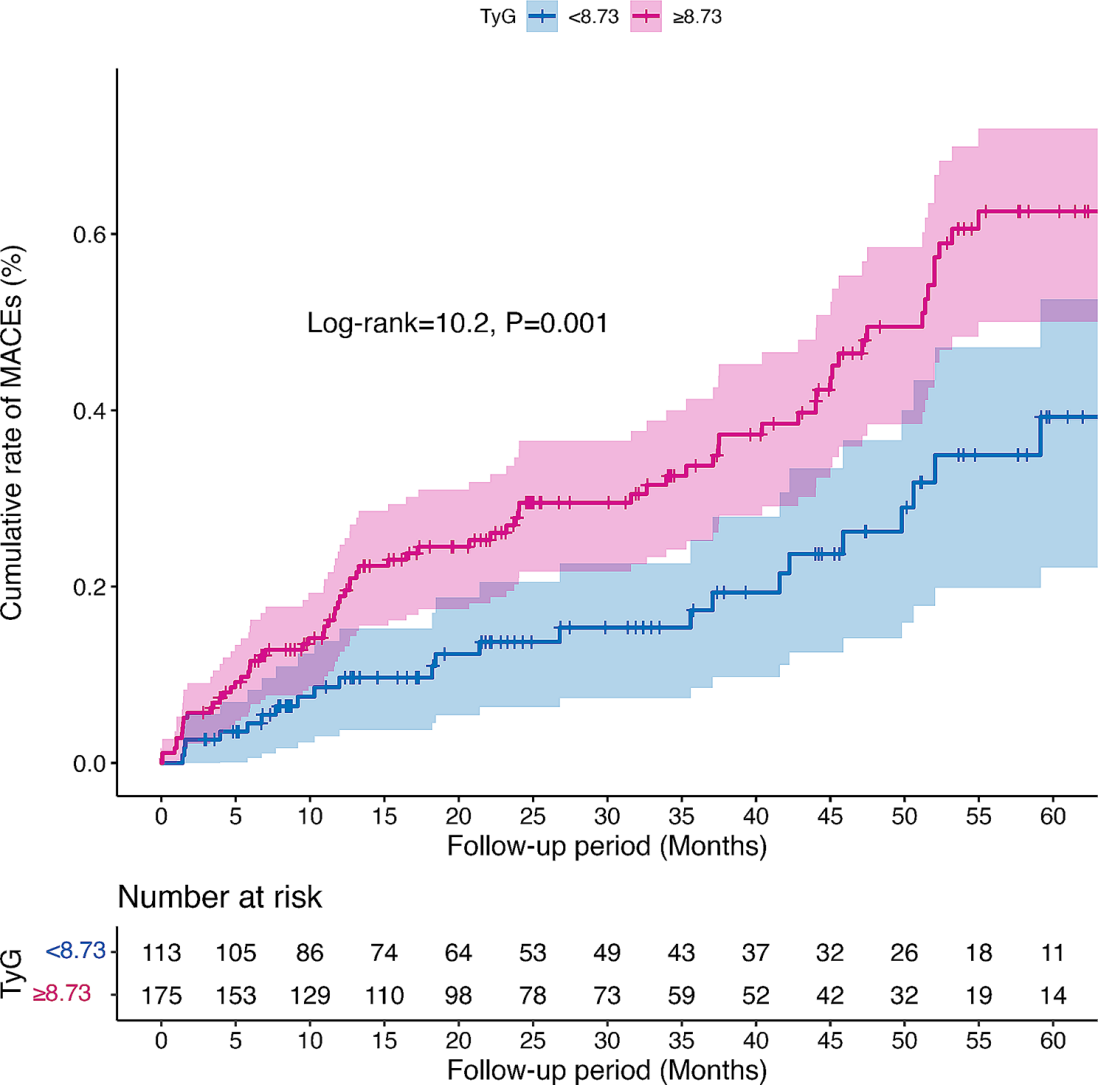
The cumulative rates of MACEs stratified by a TyG index ≥ 8.73. Abbreviations: TyG index, triglyceride-glucose index; MACEs, major adverse cardiovascular events

**Fig. 5:**
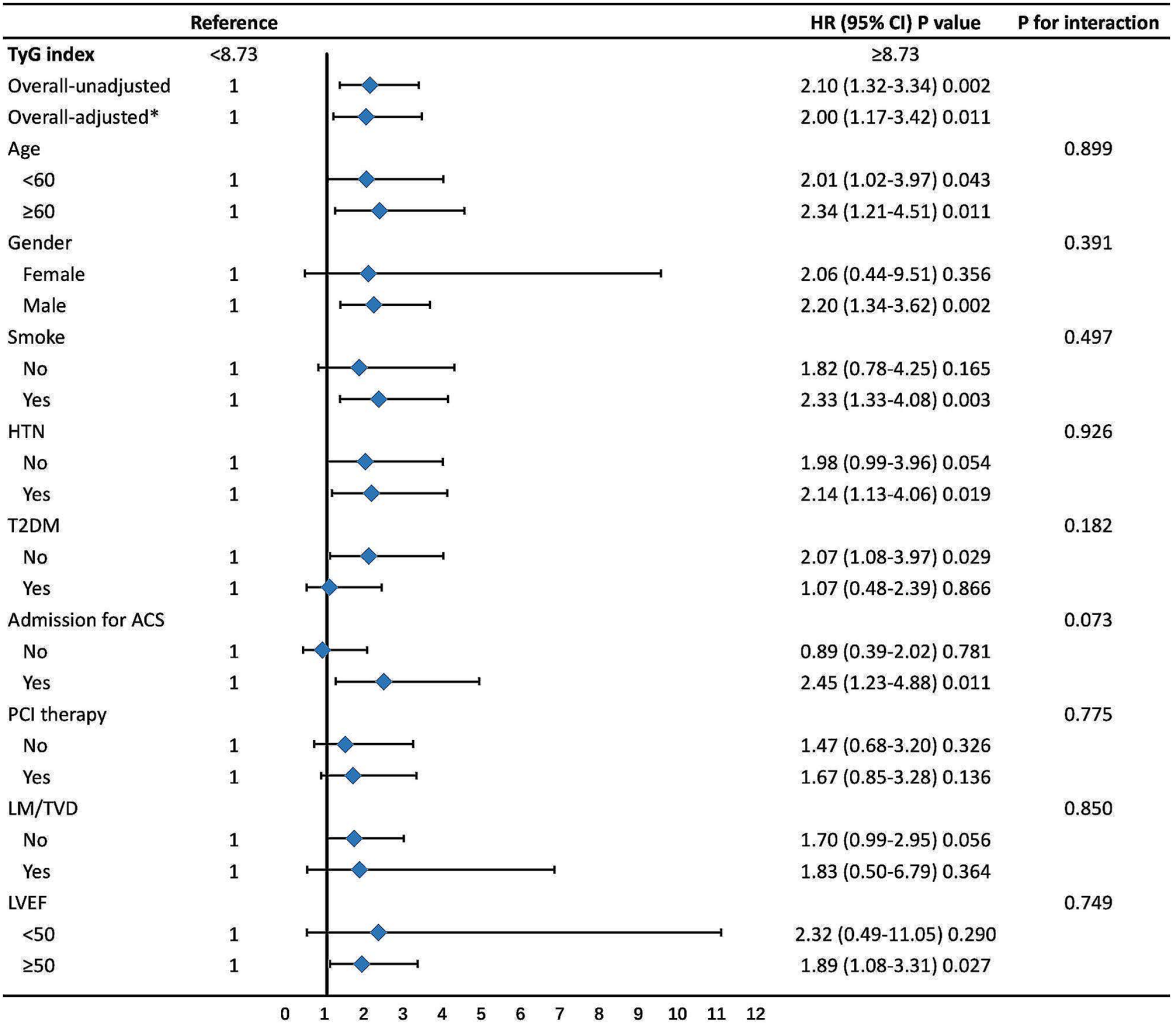
Stratified analyses of the association between TyG index and MACEs. *Adjusted for age, sex, smoking status, hypertension, T2DM, stroke, and admission for ACS, use of RAASI, platelet count, hsCRP levels, LDL-C, LVEF, presence of LM/TVD, angulated lesion, and PCI therapy. *Abbreviations*: TyG index, triglyceride-glucose index; MACEs, major adverse cardiovascular events; HTN, hypertension; T2DM, type 2 diabetes mellitus; ACS, acute coronary syndrome; RAASI, renin-angiotensin-aldosterone system inhibitors; hsCRP, high sensitivity C-reactive protein; LDL-C, low-density lipoprotein cholesterol; LVEF, left ventricular ejection fraction; LM/TVD, left main or three-vessel disease; PCI, percutaneous coronary intervention

Kaplan-Meier curve analysis demonstrated a significantly higher cumulative rate of MACE in patients with a TyG index ≥ 8.73 compared to those with a TyG index < 8.73 (log-rank test = 10.2, *P* = 0.001; Fig. 4). Furthermore, Cox regression analysis showed that patients with a TyG index ≥ 8.73 were significantly associated with a higher rate of MACE (HR = 2.10, 95% CI = 1.32–3.34, *P* = 0.002; Fig. 5). After adjustment of confounders, the TyG index remained independently associated with MACE (HR = 2.00, 95% CI = 1.17–3.42, *P* = 0.011; Fig. 5).

We then performed stratified analyses to investigate the interaction between the TyG index and potential covariates such as age, gender, smoking status, hypertension, T2DM, admission for ACS, PCI therapy, LM/TVD, and LVEF. However, the stratified analysis results did not show any interactive association between the TyG index and all the stratified variables assessed (All P interaction > 0.05; Fig. 5).

## Discussion

Both CAD and psoriasis are highly prevalent diseases worldwide. Furthermore, several studies have demonstrated a significant association between CAD and psoriasis. The co-occurrence of CAD and psoriasis may be caused by the common genetic pathways promoting inflammation in both the vascular endothelium and the skin epidermis. Previous studies have shown that the TyG index is an effective biomarker for predicting IR and inflammation and is positively correlated with poor outcomes. However, feasibility of the TyG index as a predictive biomarker in patients with both CAD and psoriasis has not been verified. To our knowledge, this is the first study to validate the TyG index as a predictive biomarker of MACE in patients with CAD and psoriasis. Our data showed that the TyG index was positively correlated with the risk of MACE in patients with both CAD and psoriasis. After adjusting for the potential confounders, the TyG index was an independent predictor of MACE in these patients. Furthermore, our data showed a linear relationship between the TyG index and MACE with a TyG index ≥ 8.73 as the optimal threshold.

IR plays a key role in the pathogenesis of T2DM [21], obesity [22], metabolic syndrome [23], and atherosclerotic cardiovascular diseases (ASCVDs) [24]. Hyperinsulinemic-euglycemic clamp test is the gold standard for assessing insulin sensitivity [25]. However, this method has restricted use in clinical settings because it is costly and time-consuming. The TyG index is estimated from the fasting triglyceride and fasting plasma glucose levels, and serves as a potential surrogate biomarker for IR [26].

Several studies have demonstrated a significant association between the TyG index and adverse outcomes across various populations. Meta-analyses of cohort studies in the general population showed that a higher TyG index was independently associated with an increased incidence of ASCVDs [27–29], non-alcoholic fatty liver disease [30], and cardiometabolic syndrome [31]. Moreover, predictive value of the TyG index has been confirmed in subjects with CAD. A higher TyG index is associated with an increased severity and complexity of CAD [32–35]. Furthermore, an higher TyG index is associated with MACE in patients with premature CAD [36], chronic coronary syndrome [37], and TVD [38], as well as in patients undergoing PCI [39], or coronary artery bypass grafting [40]. TyG index is also a significant predictor in CAD patients with comorbid conditions such as diabetes [41, 42], chronic kidney disease [43], and hypertension [44, 45]. Psoriasis is also associated with increased cardiovascular risk. Cross-sectional studies suggested that the TyG index was potentially associated with psoriasis [14] and carotid atherosclerosis in patients with psoriatic arthritis [15]. However, these studies did not establish an association between the TyG index and cardiovascular outcomes in patients with psoriasis. In this study, we demonstrated that the TyG index was positively correlated with the risk of MACE in patients with both CAD and psoriasis. Furthermore, after adjusting for potential confounders, the TyG index was an independent predictor of MACE in patients with both CAD and psoriasis.

The underlying mechanism mediating the association between the TyG index and MACE in patients with both CAD and psoriasis is unclear, but we postulate the following potential mechanisms that may mediate the relationship between the TyG index, CAD, and psoriasis.

Firstly, the co-occurrence of CAD and psoriasis is typically driven by similar biological mechanisms [46–48]. Genetic mechanisms related with chronic inflammation are involved in the clinical manifestations of both psoriasis and CAD [10, 11]. The levels of pro-inflammatory cytokines such as tumor necrosis factor-α, interleukin 1β, and interleukin 6 are elevated in psoriasis and participate in the activation of the lectin-like oxidized low-density lipoprotein receptor-1. This leads to a cascade of events that include the uptake of oxidized LDL-C by the arterial endothelial cells, monocyte adhesion, foam cell formation, and smooth muscle cell proliferation, which cause vascular stiffening and endothelial senescence [7, 49, 50]. Our previous studies also demonstrated that inflammatory biomarkers such as fibrinogen [51] and lipoprotein (a) [52] were positively associated with the poor prognosis of patients with both CAD and psoriasis. Secondly, the TyG index is correlated with IR and the degree of inflammation. Therefore, it may be an effective predictor of disease severity and adverse cardiovascular outcomes. IR is linked to endothelial dysfunction, oxidative stress, immune dysregulation, coagulation imbalance, and inflammatory responses, all of which contribute to vascular stiffness and reduced availability of nitric oxide, and increase the risk of MACE [53–60]. Cross-sectional studies by Ma et al. [61] and Yang et al. [62] demonstrated a positive correlation between the TyG index and coronary inflammation, as indicated by peri-coronary adipose tissue attenuation based on the coronary angiographic imaging results. Therefore, TyG index, an indicator for IR and inflammation, was related with the incidence of MACE in patients with both CAD and psoriasis.

It is not clear whether the association between the TyG index and cardiovascular risk is linear. Moreover, the optimal cutoff value for the TyG index to accurately predict future cardiovascular events remains unresolved. While some studies reported a U-shaped association between the TyG index and cardiovascular deaths [31, 41], several meta-analyses reported a linear relationship between the TyG index and cardiovascular risk [27–29]. Furthermore, Liang et al. performed a meta-analysis to determine relationship between the TyG index and CAD prognosis, and reported that the optimal TyG index range for predicting adverse prognosis in CAD patients was 8.3 to 9.3 [27]. Consistent with previous studies, our findings also demonstrated that the relationship between the TyG index and MACE was linear and ≥ 8.73 was the optimal threshold value for the TyG index. However, the optimal threshold value and the true relationship between the TyG index and MACE is dependent on the sample size, study population, and the specific clinical outcomes being investigated in the study. Therefore, future studies are necessary to validate our results and further establish the underlying mechanisms that mediate the relationship between the TyG index and MACE in patients with both CAD and psoriasis.

Our study has several limitations. Firstly, our study was a single-center retrospective cohort study with a small sample size. Despite adjusting for several confounding factors and the subgroup analysis results showing absence of any interaction between the variables, residual confounding may still influence the results. Secondly, PASI assessment in this study was incomplete with 53 missing values. Our analysis indicated that PASI did not statistically influence the clinical outcomes, but further investigation is necessary for confirming the results. Thirdly, our data was unable to conclusively determine whether the relationship between the TyG index and MACE was linear. Moreover, the optimal threshold value for the TyG index needs to be validated in other populations. Finally, the underlying mechanisms that link the TyG index with MACE in patients with psoriasis and CAD are not fully understood. Future studies, including randomized controlled trials in larger and more diverse populations are necessary to validate our findings and identify the underlying mechanisms.

## Conclusions

This study showed that the TyG index was positively correlated with the risk of MACE in patients with both CAD and psoriasis. The correlation remained significant after adjusting for potential confounding factors, thereby indicating that the TyG index was an independent predictor of MACE in this population. Furthermore, our study demonstrated a linear relationship between the TyG index and the occurrence of MACE. Our data showed that a TyG index of ≥ 8.73 was the optimal threshold value for predicting MACE and may have clinical utility in the early identification for the timely intervention and personalized care of patients with both CAD and psoriasis and improve their clinical outcomes.

## Data Availability

The raw data supporting the conclusions of this article will be made available by the authors, without undue reservation.

## Abbreviations

ACS: Acute Coronary Syndrome
ASCVDs: Atherosclerotic Cardiovascular Diseases
CABG: Coronary Artery Bypass Grafting
CAD: Coronary Artery Disease
HRs: Hazard Ratios
hsCRP: High Sensitivity C-Reactive Protein
IR: Insulin Resistance
LM/TVD: Left Main or Three-Vessel Disease
LVEF: Left Ventricular Ejection Fraction
LDL-C: Low-Density Lipoprotein Cholesterol
MACE: Major Adverse Cardiovascular Events
PCI: Percutaneous Coronary Intervention
PASI: Psoriasis Area and Severity Index
ROC: Receiver Operating Characteristics
RAASI: Renin-Angiotensin-Aldosterone System Inhibitors
RCS: Restricted Cubic Splines
TyG: Triglyceride-Glucose Index
T2DM: Type 2 Diabetes Mellitus
